# A change of heart

**DOI:** 10.1007/s12471-014-0567-3

**Published:** 2014-06-04

**Authors:** F. M. Zimmermann, E. van Mierlo, A. Meijer, L. R. Dekker

**Affiliations:** Department of Cardiology, Catharina Hospital Eindhoven, Michelangelolaan 2, 5623 EJ, Eindhoven, the Netherlands

## Rhythm puzzle-answer

**Fig. 2:**
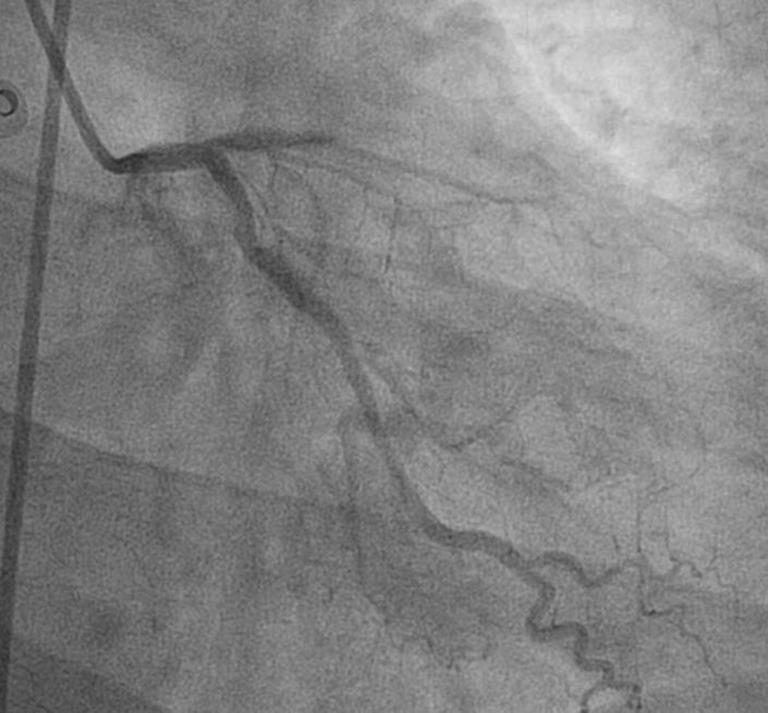
Occlusion of the mid left anterior descending artery (LAD)

The ECG shows ST elevation in leads I, II, aVL, and V1–V6 representing acute myocardial infarction of the anterolateral wall. Coronary angiogram showed an occlusion of the mid left anterior descending artery (LAD) (Fig. 2). After reperfusion it became clear that the LAD passed over the apex, explaining the ST elevations in the inferior leads.

Most notably, there is a clear ST alternans in all leads. ST alternans has been studied in several fundamental animal studies [1, 2]. Alternation of action potential duration and amplitude in the ischaemic area causes alternating variation in injury current, underlying the alternation in ST-segment elevation. This phenomenon only occurs during the first few minutes of acute ischaemia, which explains why it is rarely observed in daily practice [2, 3]. ST alternans is considered a harbinger of ventricular fibrillation [2, 3].

*Conclusion:* acute anterolateral myocardial infarction with ST alternans.
